# Histopathological Features of Pendred Syndrome Thyroids Align with Differences in the Expression of Thyroid-Specific Markers, Apical Iodide Transporters, and Ciliogenesis Process

**DOI:** 10.1007/s12022-022-09732-2

**Published:** 2022-10-15

**Authors:** V. Vázquez-Román, J. M. Cameselle-Teijeiro, J. M. Fernández-Santos, M. J. Ríos-Moreno, L. Loidi, T. Ortiz, I. Martín-Lacave

**Affiliations:** 1 Departamento de Citología e Histología Normal y Patológica, Facultad de Medicina, Universidad de Sevilla, Av. Sánchez Pizjuán S/N, 41009 Seville, Spain; 2 Department of Pathology, Galician Healthcare Service (SERGAS), School of Medicine, Clinical University Hospital, University of Santiago de Compostela, Santiago de Compostela, Spain; 3 Department of Anatomic Pathology, Virgen Macarena University Hospital, Seville, Spain; 4 Galician Public Foundation for Xenomic Medicine (SERGAS-Xunta de Galicia), Santiago de Compostela, Spain

**Keywords:** Pendred syndrome, Follicular cells, Iodide channels, Immunohistochemistry, Ciliogenesis, Primary cilia

## Abstract

Pendred syndrome (PDS) is an autosomal recessive disorder caused by mutations in the gene that encodes pendrin. Pendred thyroid tissue is supposedly altered by the absence of functional pendrin, but it is still unknown whether other iodide exchangers could compensate for the loss of the protein. Moreover, we have recently described that primary cilium, a conserved structure present at the apical surface of normal follicular cells, suffers different alterations in functional thyroid diseases. We aimed (1) to better understand the histopathological changes experienced by PDS thyroids, (2) to analyze the expression of different thyroid-specific genes and alternative iodide transporters and, finally, (3) to determine whether those changes may alter the morphological pattern of primary cilia in follicular cells. Thyroid samples from a series of four PDS patients were analyzed by immunohistochemistry, double immunofluorescence, and morphometry to evaluate changes in primary cilia frequency and length. We found thyroid follicular nodular disease in all PDS thyroids, frequently in association with follicular adenomas. There were only slight changes in the expression of thyroid-specific markers. Although no positivity for pendrin was found, cytoplasmic immunostaining for ANO-1, CLC-5, and CFTR was stronger in diffuse hyperplastic areas when compared to areas with highly cellular follicular nodules (HCFNs). HCFNs and follicular adenomas always showed diminished ciliary frequency and length. Our results suggest a direct relationship between the absence of functional pendrin and the loss of the normal thyroid architecture in PDS patients, which was also accompanied by differences in the expression of specific immunohistochemical markers and altered ciliogenesis. The present data may help the pathologist in screening for PDS.

**Supplementary Information:**

The online version contains supplementary material available at 10.1007/s12022-022-09732-2.

## Introduction

Pendred syndrome (PDS) [1] is an autosomal recessive disorder caused by mutations in the *SLC26A4* gene [2] and characterized by congenital sensorineural deafness and diffuse goiter with or without hypothyroidism [3, 4]. The *SLC26A4* gene encodes pendrin, which is mainly expressed in the thyroid gland, inner ear, and kidney [5]. In normal thyroids, pendrin is exclusively localized at the apical membrane of follicular cells [6] facing the colloid, where it functions as a Cl^−^/I^−^ exchanger involved in apical iodide efflux to the lumen [7].

Very few studies have reported the histopathological features of the PDS thyroid gland so far. Palos et al. [8], described the thyroid architecture in two patients with confirmed mutations in the *SLC26A4* gene, reporting the presence of hyperplastic changes and a lack of apical immunostaining for pendrin. Later on, Senou et al. [9] identified in one PDS patient three morphological sequential stages in the course of the disease, together with an increased expression of CLC-5 apical iodide efflux that may transiently compensate for the lack of pendrin.

We have seen in different mammals that, at the apical pole of follicular epithelium, there is at least one primary cilium (PC) per cell extending from the apex into the lumen [10]. PC has been found in numerous cell types [11], where functions as an extracellular sensory antenna associated with important signaling pathways involved in numerous cellular, physiological, and developmental processes [12]. Specifically, in the thyroid, PC, taking advantage of their ideal localization, may sense the colloid environment, contributing to the complex mechanism of thyroid hormonogenesis. Accordingly, in functional thyroid pathology, where follicular heterogeneity is exacerbated, we have detected changes in the normal ciliary pattern [13].

As we have mentioned above, pendrin is expressed at the apical pole of normal follicular cells, where PC is placed. In PDS thyroids, pendrin loss could lead to compensatory upregulation of other apical membrane iodide channels, such as CLC-5 [9, 14], ANO-1 [15], or CFTR [16], and could be related to altered ciliogenesis. To our knowledge, there is not any publication in which the presence of PC, in absence of functional pendrin, has been analyzed.

Therefore, our main objectives in the present study were as follows: first, to describe the histopathological characteristics of thyroid tissue in a more representative series of PDS patients. Second, to examine follicular cell hormonogenic status by analyzing the immunohistochemical expression of three thyroid-specific genes, thyroglobulin (Tg), TPO, and TTF-1, as well as confirming the absence of pendrin and its possible compensatory upregulation by other alternative iodide channels, such as ANO-1, CLC-5, or CFTR. Third, to analyze morphometrically whether there is altered ciliogenesis in PDS thyroid tissue, as we have previously reported for functional thyroid pathology.

## Materials and Methods

### Human Thyroid Specimens

Thyroid samples from four patients diagnosed with PDS, two hyperplastic thyroid tissues from two patients with Graves’ disease, and two normal thyroid samples were provided by the Biobank of the Department of Pathology of the Clinical University Hospital of Santiago de Compostela (CHUS), integrated in the Spanish National Biobank Network. The study complies with the guidelines for human studies and was conducted ethically in accordance with the World Medical Association Declaration of Helsinki. The protocol was approved by the Santiago-Lugo Medical Research Ethics Committee (code: 2019/275). All PDS patients suffered from deafness and three of them from goiter, and their clinicopathologic features are summarized in Table 1.

**Table 1:** Baseline clinicopathologic features of PDS patients

**Case**	**Age/sex**	**Thyroid function**	**Gene analysis** ***SLC26A4***	**Clinic symptoms**	**Histopathological findings**
PDS1*	43/female	TSH: normal T_4_: low	Mutated (compound heterozygous) c.578C → T and c.279delT	Deafness Goiter	Nodular and diffuse hyperplasia
PDS2**	26/male	TSH: high T_4_: low	Mutated (compound heterozygous) c.279delT and c.416–1G → A	Deafness No goiter	Nodular and diffuse hyperplasia
**PDS3**	30/male	TSH: normal T_4_: low	Mutated (compound heterozygous) c.279delT and c.416–1G → A	Deafness Goiter	Nodular and diffuse hyperplasia Multiple follicular adenomas 1 Oncocytic adenoma 1 Papillary microcarcinoma
**PDS4**	38/male	TSH: high T_4_: normal	Not available	Deafness Goiter	Nodular and diffuse hyperplasia Multiple follicular adenomas

^*^Patient of family A [8]

^**^Patient of family B [8]

Thyroid glands were fixed in 10% neutral buffered formalin, embedded in paraffin by standard procedure, sectioned at 4–5-µm thickness, and mounted on silane-coated glass slides. Consecutive tissue sections were stained with hematoxylin–eosin for histological diagnosis and to select appropriate thyroid tissue.

### Immunohistochemical Staining

Immunohistochemical analyses for Tg, TPO, TTF-1, and pendrin (see Table 2) were performed on paraffin sections of thyroid specimens in an immunostainer (Autostainer Link 48, Agilent, Santa Clara, CA, USA) equipped with a two-step immunohistochemical staining system (EnVision FLEX/HPR, Dako, Denmark) that uses a peroxidase-labelled polymer conjugated to the secondary antibody. Prior to immunostaining, the samples were treated for antigenic retrieval (AR) according to the manufacturer’s protocol in the pretreatment module (PT link, Dako). Immunostainings for ANO-1, CLC-5, and CFTR were assayed according to a manual procedure, using overnight incubation at 4 °C with the specific antibody, and the Vectastain ABC-HRP Kit (Vector, USA) following manufacturer’s instructions (see Table 2). Non-immune mouse and rabbit serum samples were used instead the primary antibodies as negative controls. Positive controls were included for pendrin (normal and Graves’ disease thyroid tissues); ANO-1/DOG-1 (GIST and normal gallbladder); CLC-5 (normal kidney and epididymis); and CFTR (normal gallbladder and larynx).

**Table 2:** Experimental conditions for immunohistochemistry

**Protein**	**Primary antibody**	**Incubation conditions**
**Tg**	Rabbit polyclonal (A0251, Dako, Denmark)	AR pH 6, dilution 1:2000, Immunostainer
**TPO**	Mouse monoclonal (Ab47, Dako, Denmark)	AR pH 9, dilution 1:50, Immunostainer
**TTF-1**	Mouse monoclonal (SPT24, NCL-L-TTF-1, Leica Biosystems, UK)	AR pH 9, dilution 1:50, Immunostainer
**Pendrin**	Mouse monoclonal (UIRF 01,065, MBL, Woburn, USA)	AR pH 9, dilution 1:20, Immunostainer
**ANO-1**	Mouse monoclonal (C-5, sc-377115, Santa Cruz Biotechnology, Inc, Heidelberg, Germany)	AR pH 9, dilution 1:50, overnight, ABC-HRP Kit (Vector, USA)
**CLC-5**	Rabbit polyclonal (HPA003213, Roche-Sigma, Germany)	AR pH 9, dilution 1:200, overnight, ABC-HRP Kit (Vector, USA)
**CFTR**	Mouse monoclonal (A-3, sc-376683, Santa Cruz Biotechnology, Inc, Heidelberg, Germany)	AR pH 9, dilution 1:50, overnight, ABC-HRP Kit (Vector, USA)
**Calcitonin**	Rabbit polyclonal (A-572, Dako, Denmark	Dilution 1:4000, Immunostainer

Finally, some of the sections immunostained for TTF-1 were further stained by the periodic acid-Schiff (PAS) method to verify the positivity of the colloid.

### Double Immunofluorescence Staining

Double immunostaining was carried out according to the same procedure that we have previously reported [10, 13]. In brief, after applying an antigen retrieval step, a monoclonal anti-acetylated α-tubulin antibody (Sigma-Aldrich, Germany), followed by Cy3-labelled anti-mouse IgG antibody (Jackson ImmunoResearch Laboratories, UK), was applied. Then, sections were incubated with polyclonal rabbit anti-E-cadherin antibody (Santa Cruz Biotechnology, USA), followed by Cy2-labelled anti-rabbit IgG antibody (Jackson ImmunoResearch Laboratories, UK). DAPI was added for nuclei counterstaining. Controls for specificity of the technique were performed.

The samples were observed under a fluorescence microscope (Olympus BX50) equipped with a scientific digital camera (Hamamatsu ORCA-03G). All image files were processed using Image-Pro-Plus version 7.0 software (Media Cybernetics, Rockville, USA) to create composite RGB micrographs, enhance contrast, and obtain measurements.

### Morphometrical Analysis

#### Analysis of Primary Cilia Frequency

To evaluate the frequency of PC in PDS thyroid tissue, 10–20 micrographs per case at 200 × magnification were morphometrically assessed using a software processing and image analysis (Cell* Imaging Software). In our study, zones with highly cellular follicular nodules have been termed as HCFNs, while encapsulated cellular nodules were labelled as follicular adenomas. Then, the following histological patterns were considered: (1) normal thyroid follicles; (2) microfollicles; (3) follicles exhibiting papillary infoldings or “papillary follicles”; (4) HCFNs; and, finally, follicular adenomas. The frequency of ciliated vs. non-ciliated follicular cells was assessed by analyzing the relative number of cilia protruding from the apical surface of the epithelium vs. the number of nuclei in adequately oriented sections of those thyroid follicles. In total, the presence of PC in the current study was evaluated in an average of 1900 follicular cells per case.

#### Analysis of Primary Cilia Length

PC lengths in those histological patterns were morphometrically assessed in 10–20 micrographs per case, which were acquired using Image-Pro-Plus 7.0 software with a 40 × , UPlanFl N.A. = 0.75 objective. To minimize oblique sectioned cilia length underestimation, we measured PC that were clearly well oriented towards the colloid and seemingly fully included within the 5-µm paraffin section. In brief, the length of PC was evaluated in at least 150–300 follicular cells per case, with more than 900 cilia being measured.

### Statistical Analysis

The percentage of ciliated follicular cells and cilia lengths were measured and expressed as the mean ± standard deviation. Statistical differences were tested by one-way ANOVA following the corresponding post hoc test. *P* values < 0.05 were accepted as significant.

## Results

### Different Pathological Entities Can Develop in PDS Thyroid Tissue

**Fig. 1:**
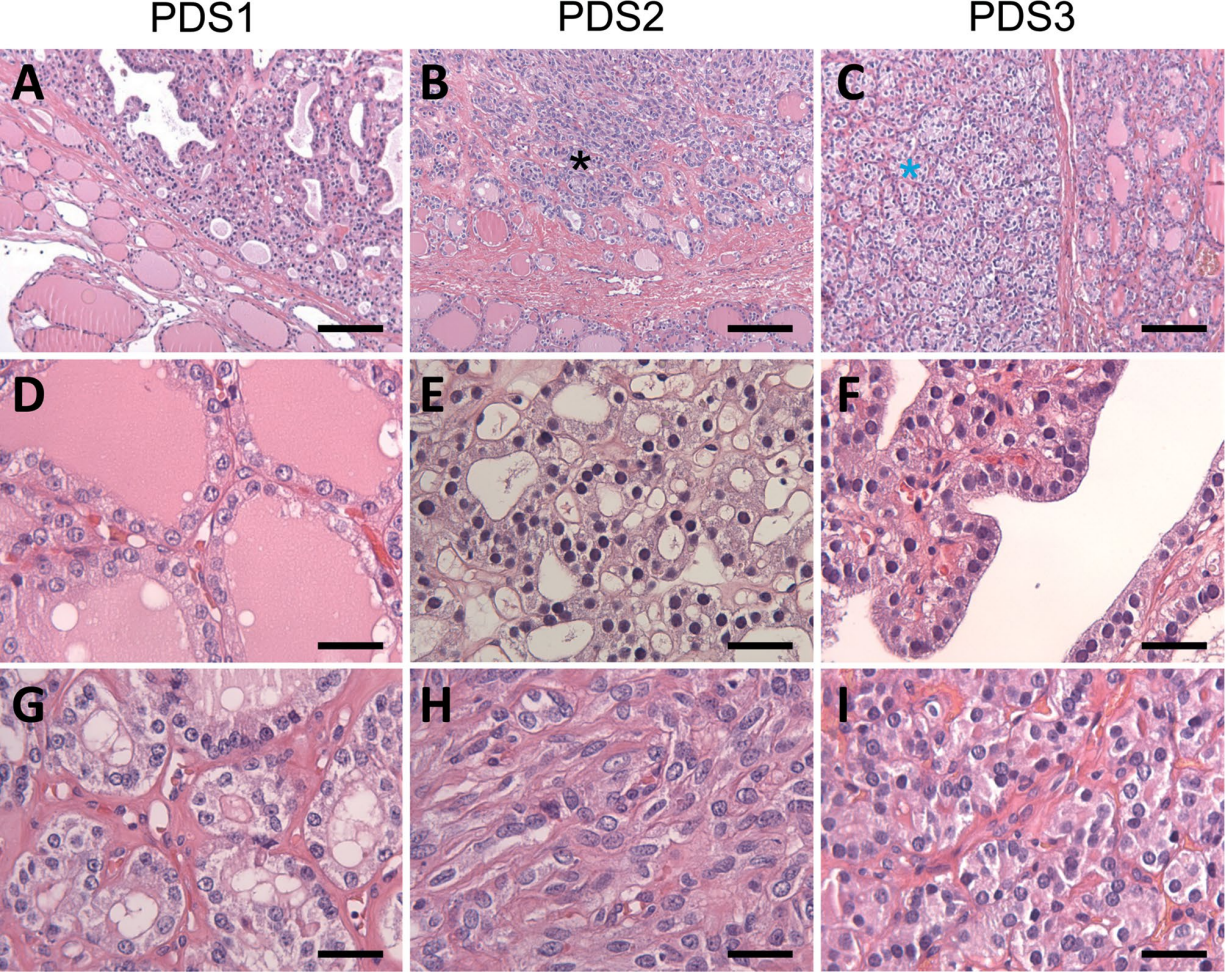
Histopathological characteristics of thyroid tissue in PDS patients stained with hematoxylin–eosin. Panoramic visions of thyroid samples in which diagnosis of nodular and diffuse thyroid hyperplasia (PDS1; **A**); diffuse thyroid hyperplasia with a highly cellular follicular nodule (HCFN) (black asterisk) (PDS2; **B**); and follicular adenoma (blue asterisk) (PDS3; **C**) were made. In amplifications of those thyroid tissues, different histologic patterns can be distinguished: **D** normal thyroid follicles; **E** microfollicles; **F** papillary follicles; **G** HCFN with microfollicular pattern and interstitial fibrosis; **H** HCFN with spindle cell features, and finally, **I** follicular adenoma exhibiting a trabecular pattern. Scale bars: 70 µm (**A**–**C**), 25 µm (**G**–**L**)

Thyroid glands from the four PDS patients were diagnosed as nodular and diffuse hyperplasia, regardless of their functional thyroid status. Multiple follicular adenomas, as well as even a subcentimeter papillary thyroid carcinoma, were observed in two of the patients (Table 1, Fig. 1A–C). The hyperplastic thyroid tissue showed areas with different follicular patterns, such as normal sized thyroid follicles (Fig. 1D), microfollicles (Fig. 1E), follicles with papillary infoldings within the lumen or “papillary follicles,” some exhibiting tall columnar cells and empty colloid in the lumen (Fig. 1F). In those areas, scattered cells with nuclear atypia and hyperchromasia were observed. Oncocytic changes of follicular cells were also seen in different follicular patterns, either as solitary or grouped cells.

Moreover, another common alteration consisted of markedly well-defined nodules with a dense cellular growth pattern (HCFNs). HCFN areas exhibited a variety of architectural appearances, which were frequently surrounded by diffuse hyperplastic thyroid tissue (Fig. 1B, black asterisk). In some cases, HCFNs presented a microfollicular pattern with abundant fibrosis and hyalinization of the stroma (Fig. 1G); in other cases, HCFNs exhibited spindle cell features (Fig. 1H). Multiple follicular adenomas were also diagnosed (Fig. 1C, blue asterisk), according to either a solid, microfollicular, or trabecular growth pattern (Fig. 1I). Although in some areas follicles with papillary infoldings were found, no true follicular thyroid adenomas with papillary architecture were observed. Additionally, extensive zones with hemorrhage and destroyed follicles were identified along different thyroid sections, altogether with abundant fibrosis. Finally, no C-cell hyperplasia was detected in our series, either in routine examination of the surgical specimens or by immunostaining of calcitonin.

### PDS Tissue Showed Slight Changes in the Expression of Thyroid-Specific Markers and Pendrin-Alternative Iodide Transporters

**Fig. 2:**
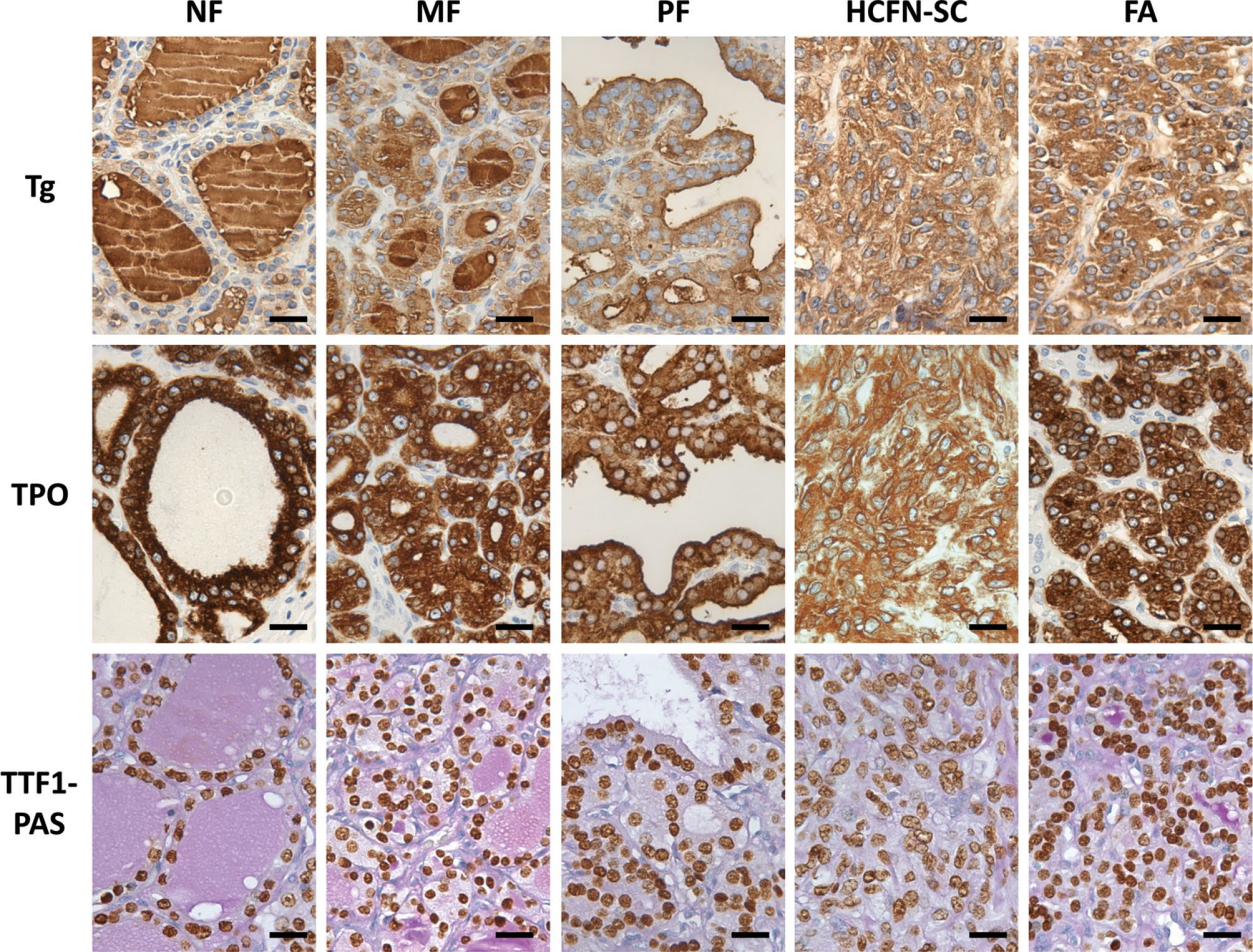
Immunohistochemical staining for thyroid-specific markers in different histological patterns of PDS thyroid tissue. Tg, thyroglobulin; TPO, thyroperoxidase; TTF1-PAS, TTF1 plus periodic acid-Schiff staining; NF, normal follicles; MF, microfollicles; PF, papillary follicles; HCFN-SC, HCFN with spindle cells; FA, follicular adenoma. No evidence of different immunostainings was observed among the histopathological patterns except for certain decrease of the positivity in HCFN with spindle cells and follicular adenoma. Scale bars, 15 µm

Immunohistochemical findings for thyroid-specific markers were rather similar in the four thyroid PDS samples (Fig. 2). In hyperplastic areas, follicular cells were immunostained for Tg, TPO, and TTF-1, independent of their histopathologic pattern although with slight differences. Specifically, in those areas of normal thyroid follicles and microfollicles, follicular epithelium was fairly stained for Tg, although with less intensity than the colloid; however, in HCFNs and trabecular adenomas, where the colloid was clearly diminished or absent, an apparent decrease of the cytoplasmic staining was observed (Fig. 2). In general, there was a direct correlation between the colloid immunopositivity for Tg and the grade of PAS intensity (Fig. 2). Moreover, no evident changes occurred for either TPO or TTF-1 expression, except when a HCFN was observed, in which immunoreactivity for both antigens was slightly diminished (Fig. 2).

**Fig. 3:**
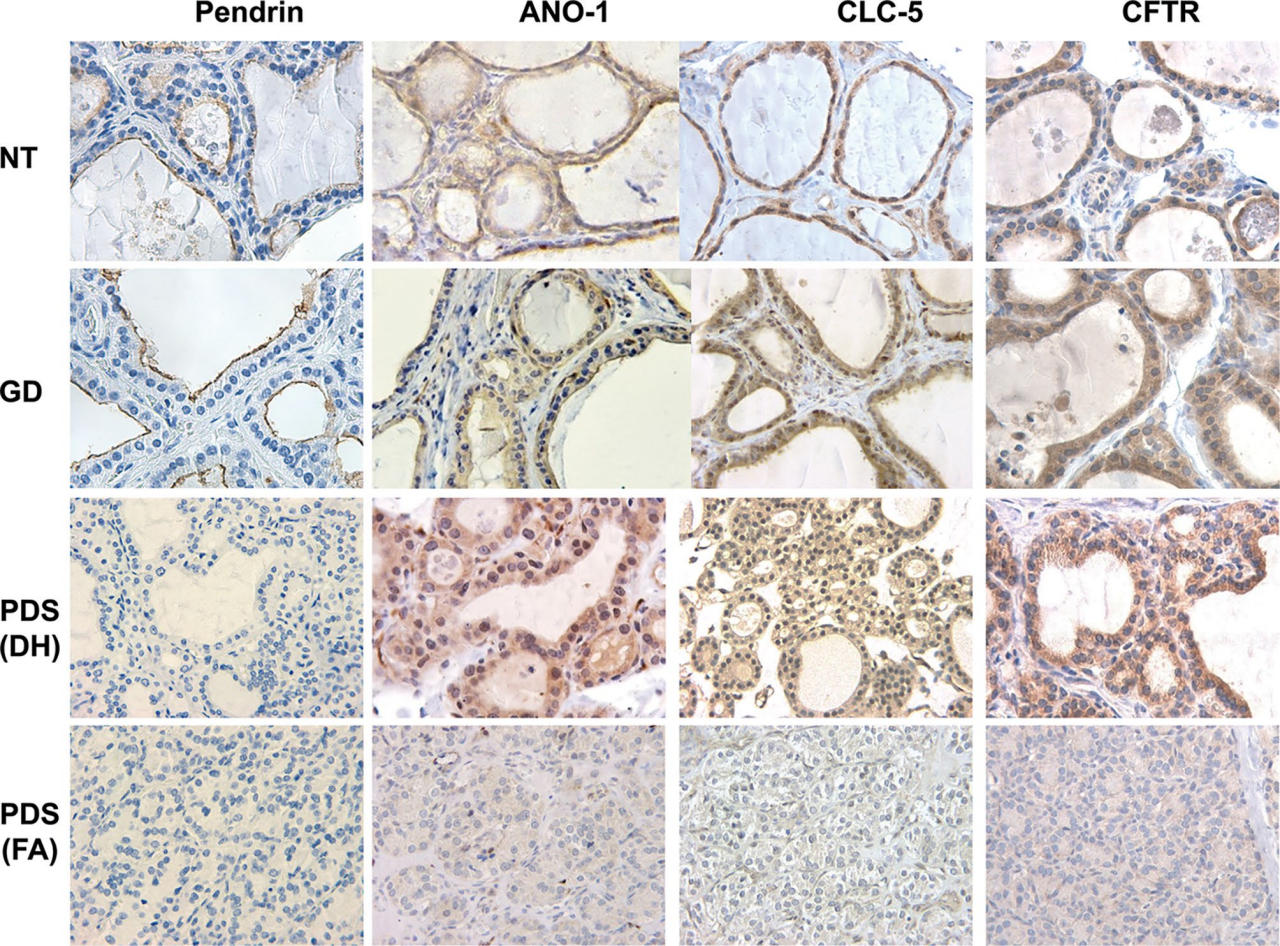
Immunohistochemical staining for several iodide transporters (pendrin, ANO-1, CLC-5, and CFTR) in controls and PDS thyroid tissues. Normal thyroid (NT), Graves’ disease (GD), diffuse hyperplastic areas (DH) and follicular adenomas (FA) of PDS patients. In contrast to NT and GD, where immunostaining for pendrin is clearly located at the apical surface of follicular epithelium, no positivity for pendrin in PDS thyroid tissue is seen. Nevertheless, immunostainings for ANO-1, CLC-5, and CFTR are positive in hyperplastic areas of PDS thyroids, as it also occurs in GD tissue, although at cytoplasmic level. Conversely, follicular adenomas are negative. Scale bars, 25 µm

Immunostaining for pendrin was completely negative in PDS thyroid tissue of the four studied patients, as expected, in contrast to normal thyroid and Graves’ disease samples that were clearly immunostained at the apical border of the follicular epithelium (Fig. 3). In relation to the expression for the alternative-iodide exchangers ANO-1, CLC-5, and CFTR, we observed immunopositivity for all of them but mainly according to a cytoplasmic pattern, although with varying intensities (Fig. 3). Specifically, in Graves’ disease thyroid tissue the staining was slightly stronger than in normal thyroids, but rather similar to that displayed by hyperplastic areas of PDS thyroid tissue. Nevertheless, in HCFNs and follicular adenomas, the expression for the three antigens was clearly decreased or even negative (Fig. 3). Positive controls for ANO-1 (gallbladder and small intestine), CLC-5 (epididymis and kidney), and CFTR (larynx and gallbladder) were clearly immunostained.

### The Frequency of Ciliated Cells and Primary Cilia Lengths Varied Among Different Areas in Accordance with Their Follicular Architecture

**Fig. 4:**
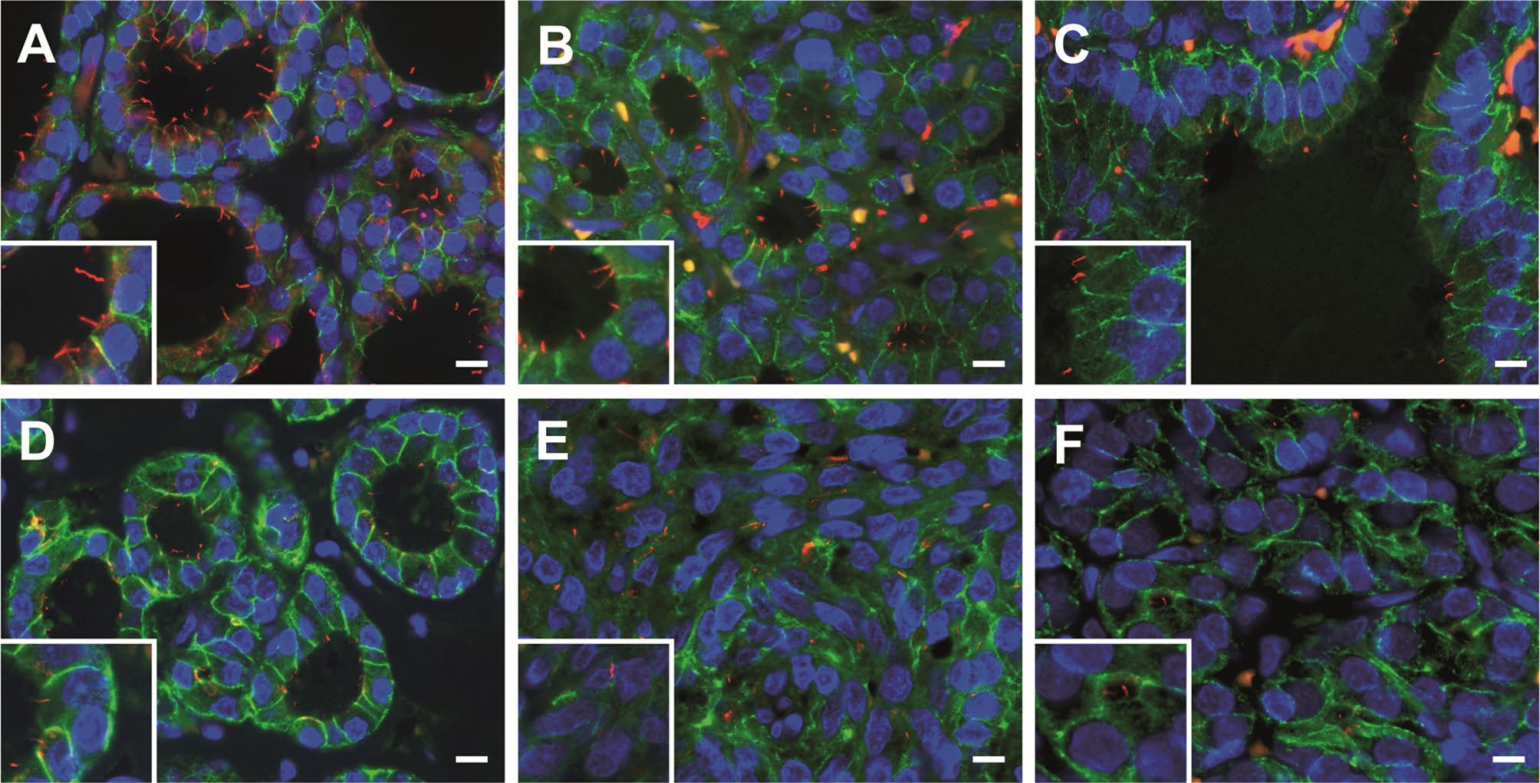
Distribution of PC in PDS thyroid tissue using double immunofluorescence (E-cadherin, green; acetylated α-tubulin, red; nuclear counterstaining with DAPI, blue). Numerous ciliated follicular cells are clearly identifiable in normal thyroid follicles (**A**), microfollicles (**B**), and papillary follicles (**C**) and much less evident in highly cellular follicular nodules (HCFNs) with microfollicular pattern (**D**). In contrast, only scarce follicular cells of HCFNs with spindle cells features (**E**) or trabecular follicular adenoma (**F**) show PC. Scale bars, 10 µm

**Fig. 5:**
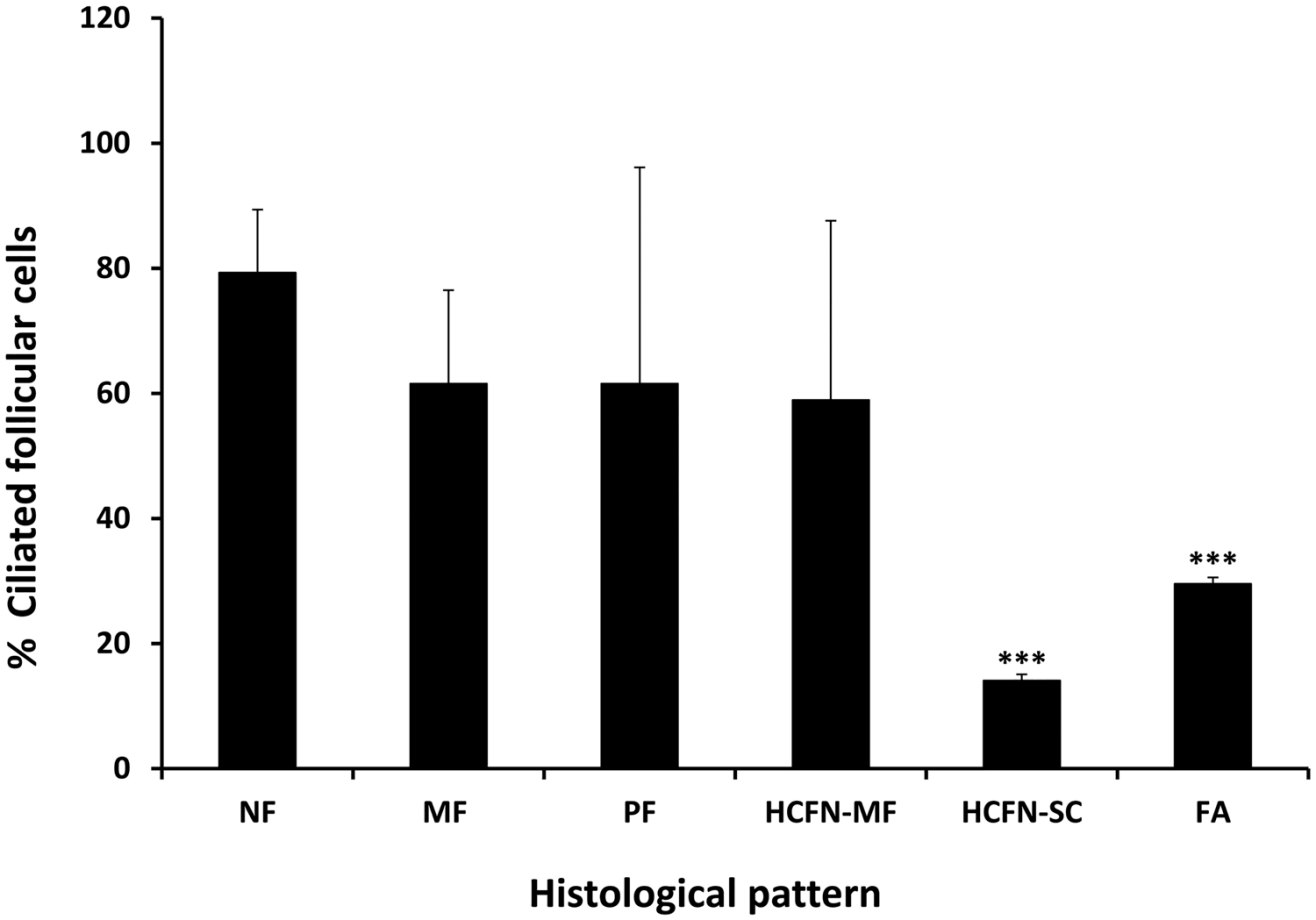
Percentage of ciliated follicular cells in the different histologic patterns observed in PDS thyroid tissue. NF, normal follicles; MF, microfollicles; PF, papillary follicles; HCFN-MF, HCFN with microfollicles; HCFN-SC, HCFN with spindle cells; FA, follicular adenoma. Results are expressed as mean ± SD. Data were compared using one-way ANOVA multiple comparisons procedures (Dunn’s method). ***P < 0.001

In general, PC emerged from the center of the apical surface of follicular cells and entered into the follicular lumen at variable angles. The frequency of ciliated cells and PC lengths varied among different areas of the samples, in accordance with their histological pattern (Fig. 4). Specifically, in the hyperplastic thyroid tissue, the highest frequency of ciliated follicular cells was found in normal follicles (79.30 ± 10.08%), followed by microfollicles (61.56 ± 34.59%) and papillary follicles (61.55 ± 14.93%), with non-significant differences. A similar number of ciliated cells was found in HCFN with microfollicular pattern (58.94 ± 28.66%), but drastically decreased either in nodules with spindle cell features (14.09 ± 1%), or in follicular adenomas (29.59 ± 1%), with statistically significant differences in the last two cases (Fig. 5).

**Fig. 6:**
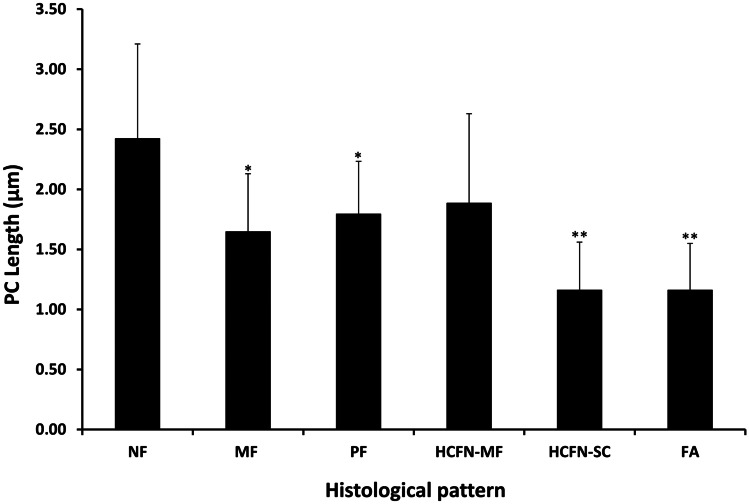
Quantitative changes in PC length among different histologic patterns observed in PDS thyroid tissue. NF, normal follicles; MF, microfollicles; PF, papillary follicles; HCFN-MF, HCFN with microfollicles; HCFN-SC, HCFN with spindle cells; FA, follicular adenoma. The differences among groups were statistically significant. The results are expressed as mean ± SD. *P < 0.05; **P < 0.01

In relation to the length of PC along the different histological areas, the longest ones were observed in normal follicles (2.4 ± 0.79), followed by HCFNs (1.89 ± 0.75), papillary follicles (1.8 ± 0.48), and microfollicles (1.65 ± 0.44), with slight statistical differences. The shortest PC was also found in both HCFNs with spindle cells (1.16 ± 0.40) and follicular adenomas (1.1 ± 0.39) (Fig. 6).

## Discussion

In our series of PDS patients, goiter was clinically present in three out of four patients. Some studies have reported that goitrous enlargement of the thyroid appears in 83% of PDS cases and can be progressive during childhood and adolescence, ultimately resulting in a multinodular goiter [3, 17]. In our series, the histopathological data fit very well with the diagnosis of “thyroid follicular nodular disease” [18]. We think this designation as opposed to adenomatoid hyperplasia or multinodular thyroid hyperplasia is more precise to define a multifocal non-inflammatory benign proliferation of follicular cells that results in multiple clonal and non-clonal nodules with highly variable architecture [18]. We also found follicular adenomas (PDS3-4) and even a papillary thyroid microcarcinoma (PDS1-2). This variable tumor progression in PDS patients is likely associated with iodide-deficient areas and prolonged overstimulation by the compensatory increased levels of TSH, carrying a 1% risk of developing thyroid carcinoma [3].

The present study confirms the results previously reported by Palos et al. [8] in two of our PDS thyroids series, and by Tong et al. in one PDS patient who eventually developed a follicular variant of papillary thyroid carcinoma [19]. According to Tong et al., the loss of pendrin functions in PDS patients represents one form of thyroid dyshormonogenesis, in which atrophic and hyperplastic changes appear together with multiple nodules of different sizes. Conversely, we did not observe the sequential progression described by Senou et al. [9] in one PDS patient by using immunohistochemical criteria, except for the existence of extensive areas of destroyed tissue. Nevertheless, we agree with them that in the absence of functional pendrin, other alternative iodide channels must take on a compensatory role. Therefore, the biosynthesis of thyroid hormones is sustained in PDS patients; hence, although half of them present hypothyroxinemia, the rest are euthyroid [17, 20].

In relation to the expression of the thyroid-specific markers Tg, TPO, and TTF-1, we observed a certain tendency of decreased immunopositivity along with the progressive loss of the characteristic follicular thyroid architecture. Moreover, when pendrin expression was studied in the four PDS thyroids, it was completely negative, as expected. In contrast, cytoplasmic immunopositive reaction for the alternative iodide exchangers ANO-1, CLC-5, and CFTR was detected in PDS thyroid tissue, being slightly increased in the areas of most hyperplastic follicles. However, none of the essayed iodide transporters was overexpressed, as it could be expected after Senou et al. [9] reported upregulation of CLC-5 in those zones that appeared histologically and histochemically normal (zone 1) in a PDS patient. Interestingly, those authors also reported that CLC-5 expression decreased in a second stage of the pathological progression of the disease (zone 2), being the iodination process interiorized in the cytosol along with increased apoptosis and cell proliferation. A similar mis-localization has been described in nodular goiter for the iodide exchanger NIS (sodium iodide symporter) that was localized in the cytoplasm but not in the baso-lateral membrane as in normal thyroid tissue [21]. Nevertheless, as Fong has suggested, thyroid iodide efflux is most likely a team effort of different anion exchangers, whose roles in iodide accumulation are complex and likely to be inter-related and orchestrated by pendrin [22].

In the same location where different anion channels are involved in mediating apical efflux for iodide organification of Tg, as a matter of fact, at the apical membrane of follicular cells, there is at least one PC per cell extending from the apex into the follicular lumen [10]. They likely function as extracellular sensory antennae that may sense the colloid environment, contributing to the complex mechanism of thyroid hormonogenesis. Therefore, in the present paper, we aimed to analyze whether, in the absence of functional pendrin, there were alterations in the ciliary pattern of PDS thyroid tissue. According to our findings, PC was easily observed in diffuse hyperplastic thyroid tissue, but the pattern changed completely when the follicular architecture evolved to HCFNs and follicular adenomas, where ciliary frequency statistically decreased. Our present data are in congruence with those recently reported by us in functional thyroid diseases [13], which suggests a direct relationship between ciliogenesis and follicle activity. Recent evidence of this relationship is the detection of some members of the anoctamin family, such as ANO-5 and ANO-10, in the apical plasma membrane of follicular cells as well as in the PC of densely grown FRT cells [23].

In contrast to recently reported C-cell hyperplasia in a case of PDS thyroid [24], we have not observed it in our series; however, our findings are not conclusive about this subject due to the limited number of paraffin blocks included in the present study.

Despite the limited number of cases in our series, we can conclude that the loss of functional pendrin in PDS thyroid tissue alters the normal thyroid architecture, which usually progress to thyroid follicular nodular disease, where HCFNs with a loss of the follicular pattern may appear. Those changes were accompanied by a progressive decrease in the frequency of ciliated follicular cells and the length of PC. Throughout this process, different iodide exchangers likely play a role to compensate altered function of pendrin. Nevertheless, to confirm if there is any relationship between ciliogenesis and apical iodide transporters, further deeper investigations are needed.

## Conclusions

Our results suggest a direct relationship between the absence of functional pendrin and the development of thyroid follicular nodular disease and, eventually, tumoral changes in the PDS thyroid architecture. Those histopathological changes were also accompanied by differences in the expression of specific immunohistochemical markers and altered ciliogenesis. Although these findings need to be confirmed in additional series, our data may help the pathologist in screening for PDS.

## Supplementary Information

Below is the link to the electronic supplementary material.

Supplementary file1 (TIF 59853 KB)

Supplementary file2 (TIF 65872 KB)

Supplementary file3 (TIF 53762 KB)

Supplementary file4 (TIF 52162 KB)

## Data Availability

The data and materials of this study are available from the corresponding author on reasonable request.
