# Supplementary figures and images for: Histopathological Features of Pendred Syndrome Thyroids Align with Differences in the Expression of Thyroid-Specific Markers, Apical Iodide Transporters, and Ciliogenesis Process
Source: Endocr Pathol. 2022 Oct 15;33(4):484–93. doi: 10.1007/s12022-022-09732-2 (PMC9712347; doi:10.1007/s12022-022-09732-2)

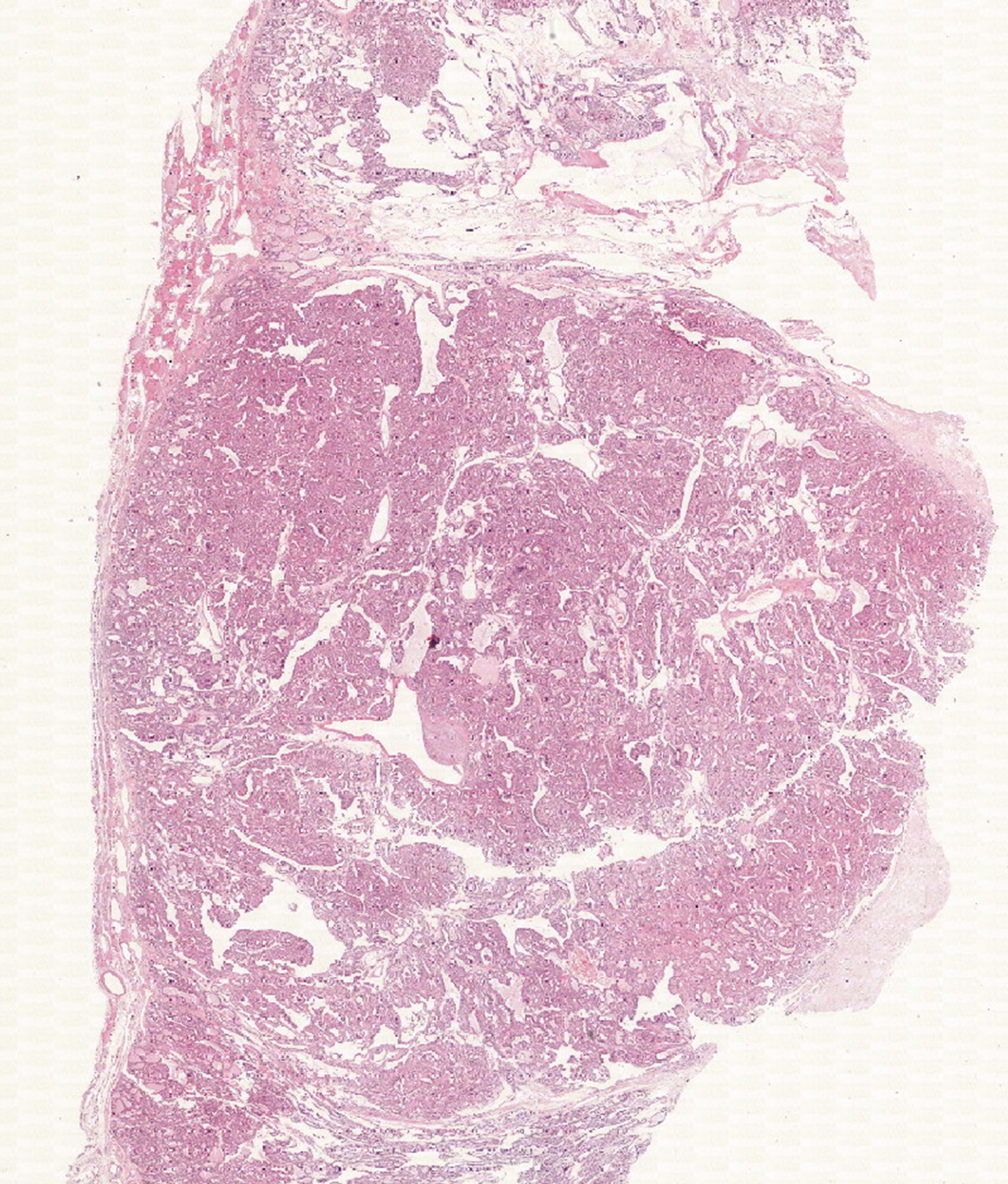

Supplement: Supplementary file 1 — Supplementary file1 (TIF 59853 KB) [file 12022_2022_9732_MOESM1_ESM.tif]

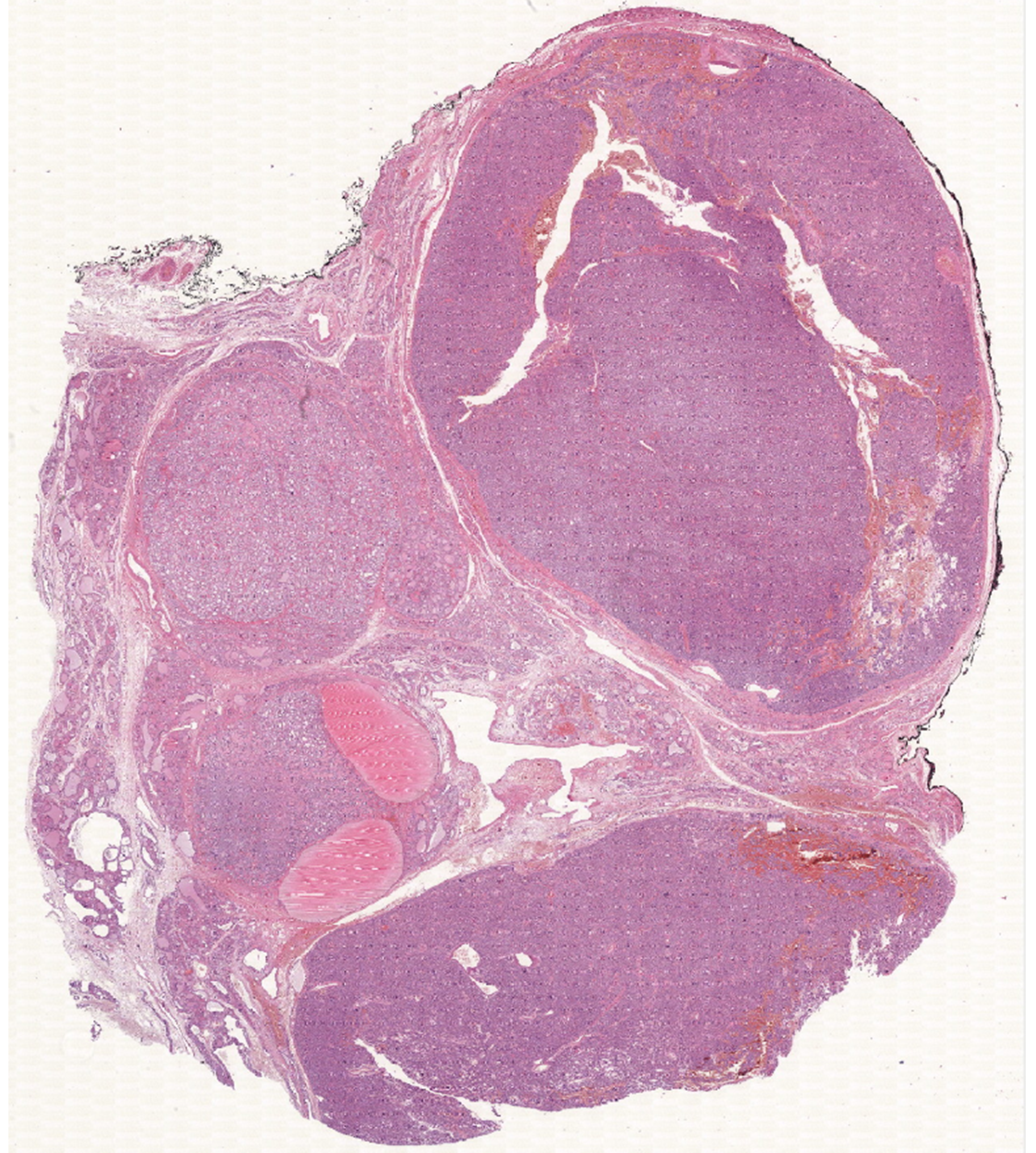

Supplement: Supplementary file 3 — Supplementary file3 (TIF 53762 KB) [file 12022_2022_9732_MOESM3_ESM.tif]

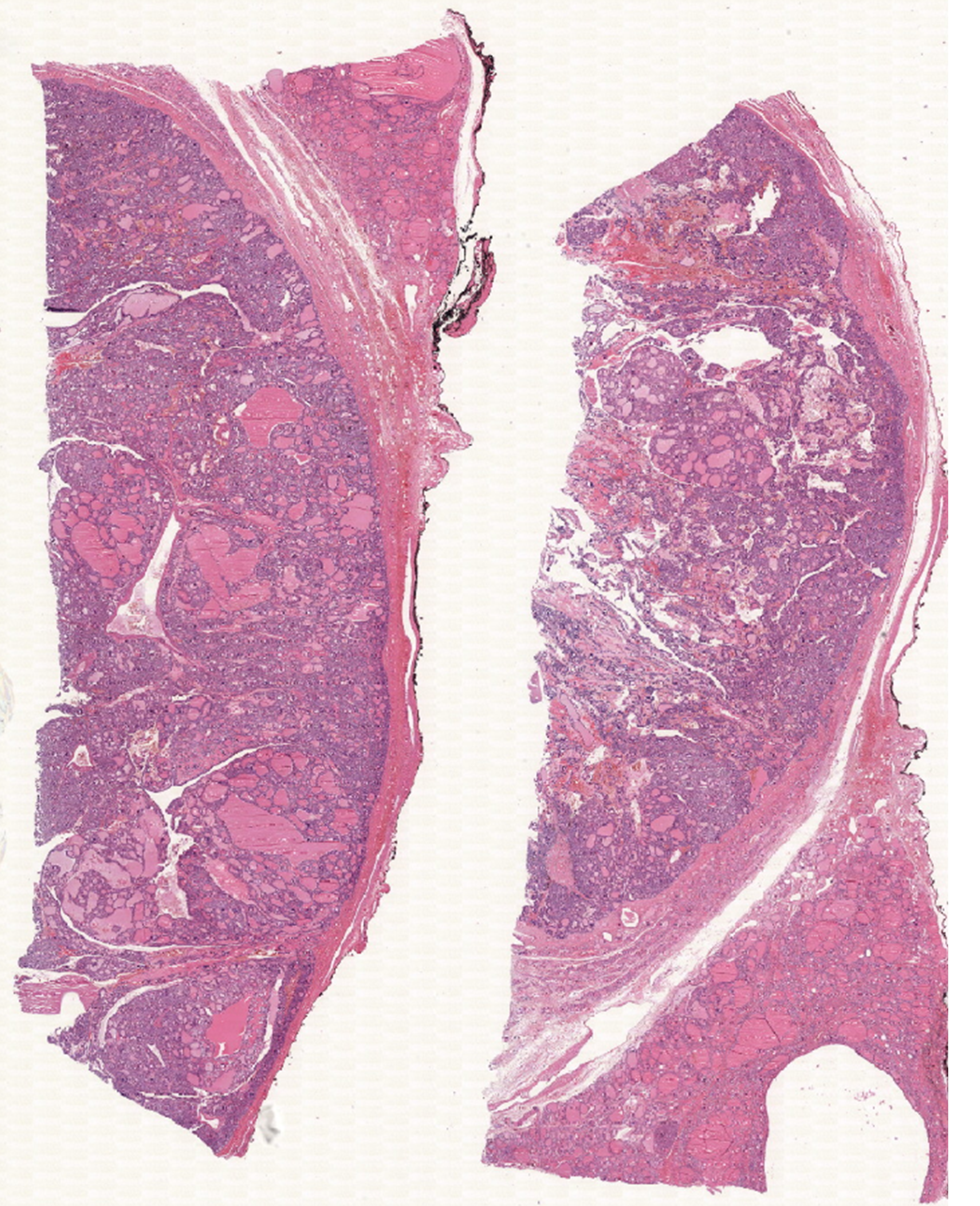

Supplement: Supplementary file 4 — Supplementary file4 (TIF 52162 KB) [file 12022_2022_9732_MOESM4_ESM.tif]
